# First person – Qingran Huo

**DOI:** 10.1242/dmm.052522

**Published:** 2025-07-07

## Abstract

First Person is a series of interviews with the first authors of a selection of papers published in Disease Models & Mechanisms, helping researchers promote themselves alongside their papers. Qingran Huo is first author on ‘ Disruption of *Morrbid* alleviates autoinflammatory osteomyelitis in *Pstpip2*-deficient mice’, published in DMM. Qingran is a graduate student in the lab of Professor Zhigang Cai at Tianjin Medical University, Tianjin, China, investigating autoinflammatory diseases.


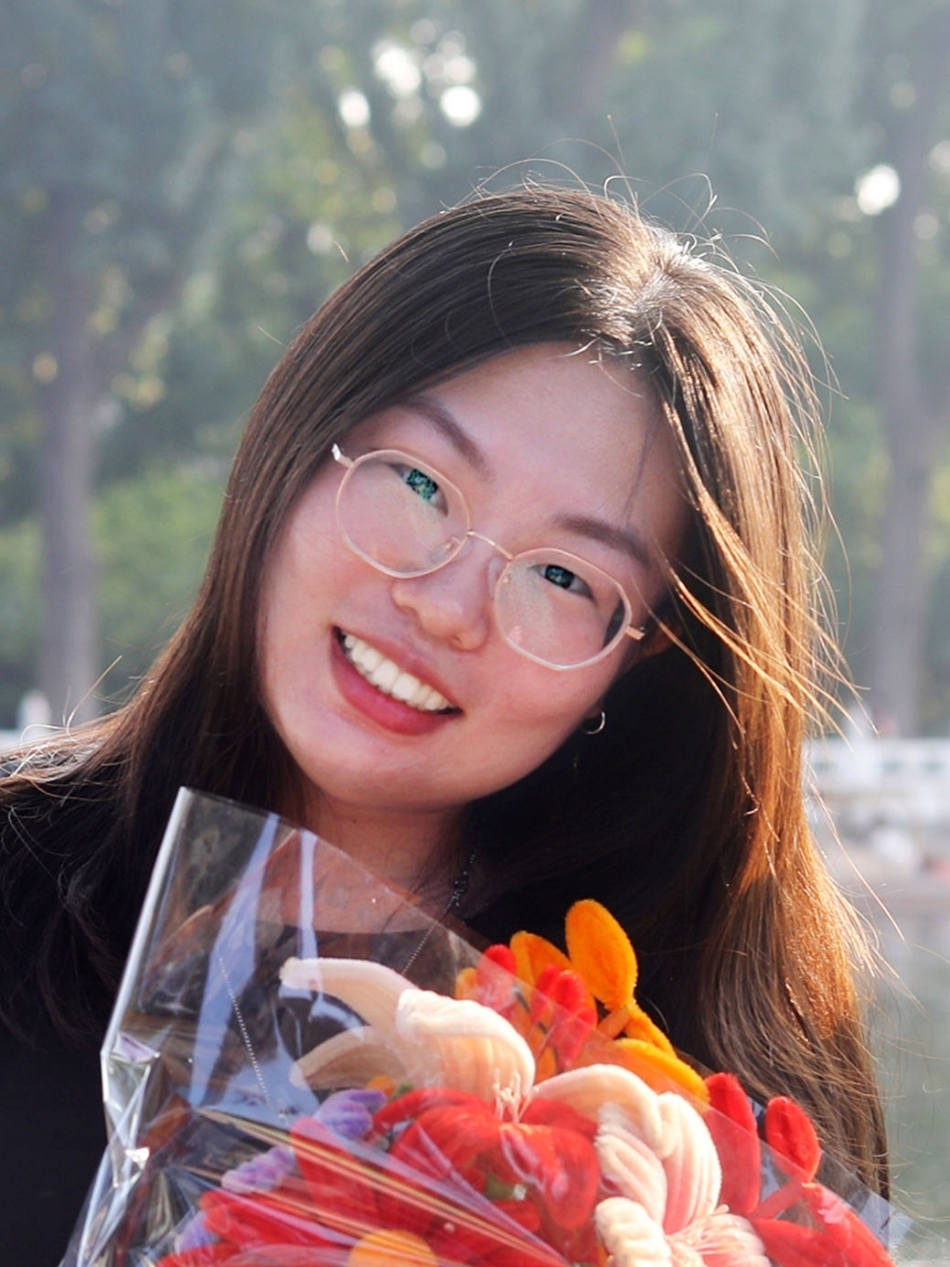


**Qingran Huo**

**Who or what inspired you to become a scientist?**

From a young age in Inner Mongolia of China, I dreamed of becoming a scientist. In my childhood, I was constantly curious about all things unknown and interesting, and eager to explore their mysteries and mechanisms. Through years of study, I gradually developed a profound interest in life sciences (the 21st century frontier), which inspired me to choose a major in medicine during my undergraduate years and continued during my postgraduate studies (starting in 2020 during the COVID-19 pandemic).

**What is the main question or challenge in disease biology you are addressing in this paper? How did you go about investigating your question or challenge?**

Autoinflammatory diseases (AIDs) are disorders caused by abnormal activation of the innate immune system. These diseases are typically closely associated with genetic factors and are clinically characterized by recurrent multi-tissue inflammation. Chronic recurrent multifocal osteomyelitis (CRMO) is one of the AIDs that affects bone tissue and causes osseous inflammation (in addition to inflammation and pain, the disease may cause patient movement disability). Its etiology remains inconclusive, and there are currently no approved drugs for its treatment. In this study, we used a mouse strain carrying *Pstpip2* mutation that mimics the phenotypic features of human CRMO. More importantly, based on this model, we introduced *Morrbid* (a long non-coding RNA co-expressed with *Pstpip2* in myeloid cells and capable of regulating the lifespan of these innate immune cells). Interestingly, although it does not encode a protein product, *Morrbid* was identified to be required for leukemogenesis in our team's previous research. Through single-cell RNA sequencing technology, a newly emerged and powerful analysis tool, we delineated the immune cell and bone marrow cell landscape of the disease, systematically revealed the etiology of CRMO, and provided a theoretical basis for targeting *Morrbid* as a therapeutic strategy for CRMO.

**How would you explain the main findings of your paper to non-scientific family and friends?**

The human body has two immunity systems – innate immunity and adaptive immunity – which serve as the host's first and second lines of defense, respectively, when pathogens invade the body. These two systems complement each other to jointly maintain the balance of homeostasis both inside and outside the body. Pathogen invasion activates the immune system and triggers inflammation. Myeloid cells, a crucial component of innate immunity, are the first immune cells recruited to the site of inflammation. When innate immunity is abnormally activated by factors other than foreign pathogens, it leads to AIDs, which are often characterized by multi-tissue involvement and recurrent episodes. CRMO, an AID affecting bone tissue, is typically marked by bone pain and can involve multiple tissues throughout the body in severe cases. CRMO affects a rare pool of patients (6.5 persons per 1 million), and its cause and prevention remain unclear. Our study not only revealed the immune cell landscape of CRMO-like mice by single-cell transcriptomic analysis, but also identified a new potential therapeutic target that may be utilized to treat or prevent the progression of this disease.

Our study [...] identified a new potential therapeutic target that may be utilized to treat or prevent the progression of this disease [CRMO]

**What are the potential implications of these results for disease biology and the possible impact on patients?**

Our results could be used as preclinical data for explaining the pathogenesis of CRMO and for developing anti-*Morrbid* antisense oligonucleotide drugs for clinical translation to inhibit CRMO. The findings will help the diagnosis and treatment of patients with CRMO and even general AIDs.

**Figure:**
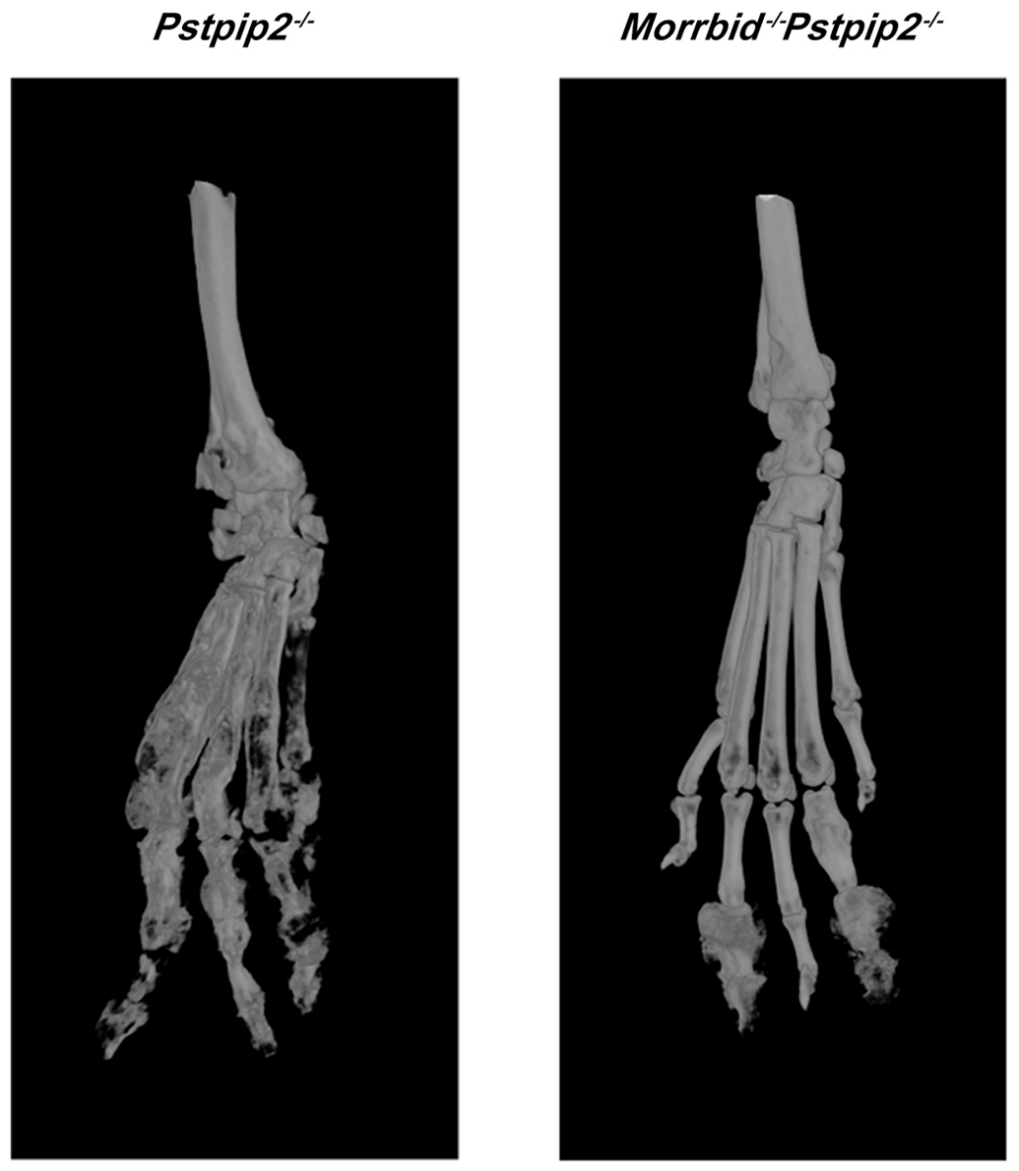
Representative micro-computed tomography of hind paw bones from 20-week-old *Pstpip2^−/−^* (left) and *Morrbid^−/−^Pstpip2^−/−^* (right) mice.

**Why did you choose DMM for your paper?**

DMM is a high-quality and well-respected journal that focuses on understanding disease mechanisms through model systems, wielding significant influence in the mechanisms, diagnosis and treatment of human diseases. My advisor, Professor Zhigang Cai, has been familiar with this journal since his postdoctoral research in mouse models in 2006 when he transferred from developmental genetics in plant models. We believe our research is highly aligned with DMM's philosophy and can provide new insights for a broad readership.

**Given your current role, what challenges do you face and what changes could improve the professional lives of other scientists in this role?**

Personally, I believe that clinical and pre-clinical research on AIDs is of great importance. This is not only because it allows us to gain a deeper understanding of the role of the immune system in diseases, but also because editing certain genes or cells within the immune system (along with bone marrow transplantations) may achieve the effect of treating or alleviating such diseases.

**What's next for you?**

In terms of this project, the next step is to conduct more in-depth research on the disease mechanisms especially the driving cell type, osteoclasts, which are a specialized type of macrophage. As for me personally, my next goal is to complete my PhD (I have just defended the thesis for my Master’s degree).

**Tell us something interesting about yourself that wouldn't be on your CV**

One of my major hobbies is music. Music gives me a lot of energy and peace. In my spare time, I enjoy finding my inner self (another me) through music.
